# Reconstructing the Tumor Mechanical Microenvironment in Organoids: From Mechanical Cues to Biomimetic Modeling and Therapeutic Insights

**DOI:** 10.7150/ijms.138880

**Published:** 2026-09-18

**Authors:** Shuying Yan, Jing Zhu, Joaquim M. Oliveira, Rui L. Reis, Leping Yan, Lin Xiao

**Affiliations:** 1 School of Biomedical Engineering, Shenzhen Campus, Sun Yat-Sen University, Shenzhen, China.; 2 Department of Chemistry, The Chinese University of Hong Kong, Shatin, N. T., Hong Kong, China.; 3 3B's Research Group, I3Bs-Research Institute on Biomaterials, Biodegradables and Biomimetics, University of Minho, Headquarters of the European Institute of Excellence on Tissue Engineering and Regenerative Medicine, Avepark - Parque de Ciência e Tecnologia, Rua Ave 1, Edifício 1 (Sede), 4805-0 17, Barco, Guimarães, Portugal.; 4 ICVS/3B's - PT Government Associate Laboratory, Braga/Guimarães, Portugal.; 5 Department of Scientific Research Center, Department of Critical Care Medicine, The Seventh Affiliated Hospital of Sun Yat-Sen University, Shenzhen, China.

**Keywords:** tumor organoids, tumor mechanical microenvironment, mechanobiology, engineered extracellular matrix

## Abstract

The tumor mechanical microenvironment (TMME) is a critical regulator of cancer initiation, progression, metastasis, and therapeutic response. Beyond biochemical signaling, physical cues such as matrix stiffness, solid stress, interstitial fluid flow, and extracellular matrix (ECM) architecture regulate tumor-cell behavior, stromal remodeling, abnormal vascular proliferation and leakage, immune interactions, and drug resistance. However, conventional *in vitro* and *in vivo* models remain limited in recapitulating the dynamic, heterogeneous, and coupled mechanical features of native tumors. Tumor organoids, particularly patient-derived tumor organoids (PDOs), have emerged as physiologically relevant and experimentally tractable platforms for modeling tumor mechanics, as they preserve key aspects of tumor architecture, cellular heterogeneity, and genetic background while enabling precise experimental manipulation and high-throughput analysis.

This review summarizes recent advances in modeling the TMME in tumor organoids. We first discuss major mechanical features of the tumor microenvironment (TME). We then discuss current strategies for recreating these features in organoid models, including stiffness-defined matrices, engineered ECMs, microfluidic platforms, mechanical actuation systems, multicellular co-culture models, and three-dimensional (3D) bioprinting. We also highlight their applications in mechanistic studies, disease modeling, and drug testing. Finally, we discuss current limitations including the overemphasis on stiffness-based design, limited reproducibility and standardization, and insufficient preservation of tumor spatial heterogeneity and mechanical gradients. We further outline future directions toward controllable, standardized, and patient-specific organoid platforms through advances in engineered matrices, microfluidics, automation, and computational analysis. By shifting from single-parameter modeling to systems-level reconstruction of the TMME, tumor organoid models are becoming increasingly powerful platforms for mechanistic discovery and therapeutic evaluation.

## 1. Introduction

Cancer remains a leading cause of death worldwide. Despite major advances in early detection, diagnosis and treatment, durable disease control remains limited for many solid malignancies 1. This gap reflects, at least in part, an incomplete understanding of how tumor cells interact with and adapt to their surrounding microenvironment during tumor initiation, progression and therapeutic response. Accordingly, the tumor microenvironment (TME) has shifted from being viewed as a passive backdrop to being recognized as a central determinant of cancer biology 2.

The TME is a dynamic and highly heterogeneous ecosystem composed of malignant cells, stromal cells, immune cells and extracellular matrix (ECM). These components collectively influence tumor progression and treatment resistance 3. Although cancer research has traditionally emphasized biochemical signaling and genetic alterations, it is now increasingly clear that the tumor mechanical microenvironment (TMME) is an equally fundamental dimension of the TME. Mechanical abnormalities in tumors, including growth-induced solid stress, matrix stiffness, altered viscoelasticity, elevated interstitial fluid pressure (IFP) and flow shear stress, are not merely by-products of tumor expansion, but active regulators of malignant progression 4-6. Through mechanotransduction pathways, these physical cues reshape cell adhesion, polarity, migration, stemness, metabolism, transcriptional programmes and drug sensitivity, thereby promoting tumor growth, vascular and lymphatic dysfunction, invasion, metastatic dissemination, immune escape and therapy resistance 5, 7, 8.

A major challenge is that the TMME is highly dynamic, spatially heterogeneous and tightly coupled to tissue architecture. For instance, during tumor progression, ECM remodeling driven by collagen deposition and crosslinking not only leads to progressive stiffening but also establishes spatial stiffness gradient 4, 9, 10. However, these features are challenging to faithfully recapitulate in conventional experimental platforms, including two-dimensional (2D) cell cultures and *in vivo* animal models. 2D cultures fail to reproduce the three-dimensional (3D) architecture and mechanical context of native tumor 11, 12, whereas animal models are limited by interspecies differences, high cost and low throughput 11, 13. These limitations have driven the development of biomimetic *in vitro* platforms that can more faithfully reconstruct TMME and thereby improve both mechanistic studies and translational discovery 13.

In this context, the new approach methodologies (NAMs) have been proposed to provide more physiologically relevant alternatives while adhering to the 3R principles (Replacement, Reduction, and Refinement) 14, 15. Among them, patient-derived organoids (PDOs) have emerged as a promising NAMs platform. Organoids are miniature *in vitro* tissue models that arise from the self-organization of stem cells or tissue-resident progenitor cells under 3D culture conditions, recapitulating key structural and functional attributes of their native organs. PDOs, in particular, are generated from tumor cells dissociated from patient specimens; while faithfully preserving the genetic, phenotypic, and functional heterogeneity of the original malignancy 16. They effectively preserve the histological features, molecular traits and intratumoural heterogeneity of the original tumor, while retaining the scalability and experimental accessibility of *in vitro* culture 17-20. Importantly, PDOs are highly compatible with engineering strategies for reconstructing the TMME. Organoids can recapitulate matrix stiffness, viscoelasticity, interstitial flow and shear stress, enabling systematic investigation of tumor mechanobiology 10, 21. Likewise, the integration with dynamical systems introduces controlled interstitial flow, pressure gradients and shear stress, thereby extending tumor organoids from static 3D cultures to more physiologically relevant dynamical models 22, 23. Recent studies further demonstrate that tumor ECM-mimetic hydrogels and engineered matrices can directly modulate chemoresistance and phenotypic plasticity in PDO models 24.

Despite this rapid progress, the field remains fragmented. Existing studies often focus on isolated mechanical variables, employ distinct material systems or device designs, and use heterogeneous readouts to assess biological responses. Consequently, it remains difficult to obtain a coherent view of which mechanical features of the TME can currently be modeled in tumor organoids, how these features are engineered and measured, and what biological insights they provide. The convergence of biomaterials science, microfabrication, mechanobiology and multi-omics further underscores the need for a timely synthesis of recent advances, technical bottlenecks and priorities for developing more physiologically relevant and reproducible tumor organoid models.

In this review, we examine how tumor organoids are being developed as biomimetic platforms for modeling the TMME. We first summarize the major mechanical features of the TME and their roles in cancer progression. We then discuss current strategies for reconstructing these cues in organoid systems, focusing on stiffness-defined matrices, engineered extracellular matrices, microfluidic systems, mechanical actuation platforms, multicellular co-culture models and 3D bioprinting. We further highlight their applications in mechanistic studies, disease modelling and therapeutic testing, followed by a discussion of current limitations and future directions. By bridging cancer biology with mechanobiology and bioengineering, tumor organoids provide a powerful framework for elucidating the mechanical regulation of cancer.

## 2. Literature Search Strategy and Evidence Evaluation

To contextualize recent advances in this field, this narrative review used a targeted search strategy to identify relevant literature. Literature searches were performed in PubMed, Web of Science, and Google Scholar using combinations of keywords including “tumor mechanical microenvironment” “cancer mechanobiology”, “matrix stiffness”, “YAP/TAZ”, “integrin signaling”, “hydrogel”, “mechanotransduction”, “tumor organoid”, “patient-derived organoid”, “bioprinting”, “microfluidics”, “tumor-on-a-chip”, and “3D culture”. Publications from 2020 to 2026 were prioritized, and additional relevant studies were identified by screening references from key articles and recent reviews.

Studies were selected based on their relevance to tumor mechanics, mechanotransduction, and the development of advanced *in vitro* tumor PDO models. Original research articles were prioritized, while selected reviews were included to provide broader conceptual and methodological context. Studies with no direct relevance to tumor mechanics or insufficient experimental evidence were excluded. The included studies were further considered according to evidence type, biological or mechanistic validation, and reproducibility to inform the assessment presented in the subsequent tables. This structured methodology serves as the foundation for the narrative synthesis presented in this article.

As part of the evidence evaluation, validation status was categorized according to the level of biological and mechanistic validation. “Biologically validated” refers to models supported by experimental evidence demonstrating relevant biological phenotypes or responses, whereas “Mechanistically validated” indicates models in which specific mechanical-biological relationships have been experimentally investigated.

## 3. The TMME as a Driver of Cancer Progression

### 3.1 Dynamic Evolution of the TMME During Tumor Progression

The TMME comprises extracellular physical cues within tumor tissues, including solid stress 9, matrix stiffness 25, viscoelasticity, IFP 26, and fluid shear stress 27. These mechanical features evolve continuously during tumor progression rather than remaining static, thereby generating a dynamic physical landscape that actively contributes to malignant progression (**Figure 1**) 28.

During the early stage of tumorigenesis and *in situ* growth, tumor cells proliferate rapidly within a confined space. This leads to the accumulation of solid stress within the tumor 9, 29. This results in compression and densification of ECM fibers, a progressive increase in tissue stiffness, and alterations in viscoelastic properties 30. Concurrently, mechanical cues activate the YAP/TAZ, FAK, and Rho/ROCK signaling axes in tumor cells via integrin-mediated mechanotransduction. These pathways act synergistically to promote cell cycle progression, enhance cellular contractility and adhesion, and inhibit apoptosis, thereby further improving cell survival and proliferative capacity.

As tumor volume increases, rapidly proliferating cells compress the surrounding vessels and lymphatic vasculature 29. The mechanical compression of blood vessels leads to insufficient perfusion and induces local hypoxia. The hypoxic microenvironment further stimulates aberrant angiogenesis 31, 32. However, the newly formed vessels are structurally immature and exhibit high permeability 33-35. These pathological features lead to persistent vascular leakage, enabling continuous extravasation of plasma components into the tumor interstitium. Under sustained compression, lymphatic vessels undergo collapse or functional impairment, resulting in impaired interstitial fluid drainage 36, 37. Due to increased fluid influx and restricted efflux, interstitial fluid progressively accumulates within the tumor tissue, leading to a sustained elevation of tumor IFP. The elevated interstitial pressure further disrupts local blood flow distribution 38, resulting in heterogeneous flow velocities and aberrant fluid shear stresses. These mechanical stimuli continuously promote aberrant vascular proliferation 39, 40, thereby establishing a self-amplifying pathological feedback loop.

As the tumor evolves, local stress relaxation induces reorganization of collagen fibers at the tumor margin. The fibrous network is progressively straightened and remodeled into a highly aligned, anisotropic structure 41. During this process, cancer-associated fibroblasts (CAFs) further facilitate collagen fiber alignment through sustained application of traction forces and contractile activity 42. In the meantime, matrix metalloproteinase (MMP)-mediated ECM degradation increases matrix porosity. These mechanical and biochemical cues collectively drive ECM remodeling at the tumor margin, transforming it from a restrictive barrier into a permissive conduit for cell migration 43. This structural reorganization thereby provides a physical framework that facilitates tumor cell outward invasion. The remodeling of the edge ECM creates a distinct stiffness gradient between the stiffened ECM and the tumor core. This promotes durotactic migration, whereby tumor cells breach the basement membrane (BM) and invade the surrounding tissue 44.

In addition, at the advanced stage of tumor development, elevated IFP drives the formation of interstitial fluid flow. This flow directly influences cellular behavior by applying mechanical shear stress to cells, and it also serves as a transport medium for various soluble signaling factors, thereby further enhancing the invasive capacity of tumor cells 45, 46. Ultimately, under the combined effects of ECM remodeling and interstFitial fluid flow, tumor cells migrate along directionally aligned fiber structures, breach the BM and invade the surrounding tissue 47. The migrating cells further remodel the ECM, establishing a positive feedback loop that promotes tumor invasion. Overall, the TMME undergoes a dynamic, stage-dependent evolution, during which distinct physical cues emerge (**Figure 2**). These cues are subsequently converted into intracellular mechanotransduction signals and cellular responses that together promote cancer progression.

### 3.2 Mechanisms by Which the TMME Drives Cancer Progression

#### 3.2.1 Reprogramming of Tumor Proliferation Driven by Solid Stress Accumulation

During the incipient stages of tumorigenesis, the rapid expansion of neoplastic cells within confined anatomical compartments generates mechanical antagonism with the surrounding parenchyma, leading to the progressive accumulation of solid stress. Mounting evidence suggests that this mechanical loading is not merely a passive byproduct of growth but serves as a pivotal determinant orchestrating cell fate 48. Specifically, elevated ECM stiffness activates integrin-dependent focal adhesion kinase (FAK) signaling and downstream Rho/ROCK pathways 49, which synergize with the Ras-MAPK-ERK 50 and PI3K-Akt-mTOR axes to accelerate cell cycle progression (**Figure 3A**) 51. Simultaneously, increased matrix rigidity inhibits the activity of LATS1/2 kinases within the Hippo pathway 52, inducing the dephosphorylation and nuclear translocation of YAP/TAZ. Once in the nucleus, these co-activators bind to TEAD transcription factors to drive the expression of proliferation-related genes, such as Cyclin D1, thereby amplifying the growth-promoting effects of mechanical signals 53, 54.

Recent studies further indicate that YAP/TAZ not only respond to ECM stiffness and cellular traction forces but also maintain their activity under hypoxic and high-pressure conditions, thereby suppressing apoptosis and promoting sustained proliferation. As a critical hub integrating mechanical cues with metabolic reprogramming, YAP/TAZ regulate enhanced glycolysis, lipid synthesis, and redox homeostasis, supporting the adaptive growth of tumor cells 55. Furthermore, mechanical stress modulates signaling pathways such as MAPK through mechanosensitive ion channels like PIEZO1 and cytoskeletal reorganization 56, establishing a positive feedback loop with canonical oncogenic pathways like Wnt/β-catenin to reinforce the proliferative advantage of tumor cells 57. Consequently, during early tumor development, solid stress accumulation drives sustained proliferative capacity through a continuous "mechanosensing-signal transduction-metabolic reprogramming" cascade, laying the foundation for subsequent structural remodeling and invasive transformation.

#### 3.2.2 ECM Remodeling and Structural-Functional Dysfunction of Vessels and Lymphatics

Under physiological conditions, blood and lymphatic vessels cooperate to maintain tissue fluid balance and support normal metabolic exchange 58, 59. However, during the intermediate stages of tumor progression, the increasing tumor volume and accumulated solid stress exert significant mechanical compression on the intratumoral vascular and lymphatic systems, resulting in systemic perfusion and drainage failure. In the vasculature, persistently elevated matrix stiffness and solid stress act directly on endothelial cells via the integrin-FAK-ERK and RhoA/ROCK-mediated contractile axes, disrupting cytoskeletal tension homeostasis and inducing vascular collapse and pathologically increased permeability 60. Concurrently, the KLF2/PIEZO1 mechanosensing axis in the endothelium becomes dysregulated under abnormal shear and compressive stresses, impairing vascular homeostasis and exacerbating perfusion heterogeneity 56.

In the lymphatic system, ECM stiffening weakens the stretching and pumping functions of lymphatic endothelial cells by inhibiting the mechanical response of the VEGFR3-PI3K/AKT signaling axis and inducing YAP/TAZ-dependent transcriptional abnormalities 61, 62. Furthermore, sustained matrix compression interferes with lymphatic differentiation and maintenance by suppressing PROX1-related transcriptional networks, leading to structural collapse and impaired interstitial fluid drainage 63. Notably, mechanosensitive channels like PIEZO1 play a critical role in both vascular and lymphatic endothelia; their sustained activation under abnormal mechanical tension induces aberrant calcium influx, amplifying RhoA/ROCK-mediated contraction and reinforcing luminal closure (**Figure 3B**) 64, 65. Ultimately, these dysfunctions culminate in significantly elevated IFP, which further inhibits perfusion and drainage by reducing the transvascular pressure gradient, creating a vicious mechanical cycle.

#### 3.2.3 Invasion and Migratory Remodeling Driven by Mechanical Gradients

In the advanced stages of tumor progression, the tumor periphery evolves into a highly heterogeneous mechanical interface. Driven by continuous ECM remodeling and traction forces mediated by CAFs, spatially distinct mechanical gradients are established 66. These gradients, alongside collagen fiber alignment, constitute a directional mechanical signaling system that drives invasion.

During ECM remodeling, CAFs activate FAK through integrin clustering, subsequently recruiting Src family kinases and p130Cas to drive Rac1-mediated leading-edge extension and RhoA/ROCK-mediated contractile tension (**Figure 3C**) 67, 68. This molecular cascade facilitates the spatial polarization of traction forces and the realignment of ECM fibers. High IFP acts as a key physical driver, inducing directional interstitial flow via pressure gradients; the resulting shear stress reorganizes disordered collagen fibers into parallel "contact guidance" tracks, providing physical "highways" for cell migration 69, 70. Additionally, IFP-induced flow transports autocrine/paracrine chemokines (e.g., CCL21) toward the tumor periphery, guiding chemotactic migration 71, 72. At the BM, mechanical stress synergizes with YAP/TAZ, β-catenin, and AP-1 to upregulate MMPs 73, leading to the structural degradation of the BM and the formation of local invasive tracks 74.

During migration, cells exhibit classic durotaxis, preferentially moving toward regions of higher ECM stiffness 75. This process relies on the FAK-RhoA-ROCK axis to establish front-rear mechanical asymmetry, with continuous mechanical feedback reinforcing migratory stability. Furthermore, mechanical stimuli trigger the epithelial-mesenchymal transition (EMT), enhancing cellular plasticity and deformability—characterized by E-cadherin downregulation and vimentin upregulation—to facilitate transit through confined extracellular spaces 48, 76.

#### 3.2.4 Synergistic Enhancement of Mechanical Barriers and Immune Escape

Concurrent with tumor cell migration is the phenomenon of immune evasion. The TMME actively contributes to immune evasion by combining physical exclusion with mechanobiological suppression of anti-tumor immunity 77. As a result, it has emerged as an important determinant of immunotherapy resistance.

Physical barriers are a major component of this effect 78. Dense and highly crosslinked ECM further limits immune-cell migration through steric hindrance, producing an effective “molecular sieve” that impairs T-cell entry and trafficking 79, 80. Recent studies indicate that LOX-mediated collagen crosslinking not only increases stiffness but also significantly reduces the effective migration velocity of immune cells within the stroma 81. Elevated IFP, together with impaired lymphatic drainage and vascular compression, reduces convective transport within the tumor interstitium and restricts infiltration of immune effector cells 9, 77, 79, 82. In parallel, vascular compression exacerbates hypoxia and metabolic stress, further reinforcing an immunosuppressive tumor milieu 9.

Beyond physical exclusion, mechanical cues directly alter immune-cell and tumor-cell signaling. Rigorous ECM-induced mechanotransduction activates YAP/TAZ signaling within tumor cells, which not only drives metabolic adaptation but also directly upregulates immune checkpoints such as PD-L1 (**Figure 3D**)^83^, ^84^. Furthermore, mechanical stimuli exert multidimensional inhibitory effects on T-cell effector functions. For example, activation of the mechanosensitive ion channel PIEZO1 in T cells has been shown to trigger the GRHL3-RNF114 axis, promote F-actin degradation and reduce cytotoxic force generation, thereby contributing to resistance to anti-PD-1 therapy 85. Additionally, the phenotypic polarization and inflammatory functions of dendritic cells (DCs) exhibit high mechanosensitivity; matrix stiffness precisely modulates DC antigen-presenting efficiency through Hippo signaling and Piezo1-mediated calcium influx 86-88.

Mechanical remodeling of the TME also indirectly shapes immune suppression by promoting hypoxia and stromal activation. Hypoxia-driven HIF-1α signaling enhances the production of immunosuppressive cytokines, including IL-10 and TGF-β, and promotes macrophage polarization towards an M2-like phenotype 89, 90. Hypoxic signaling further supports the recruitment of regulatory T cells and myeloid-derived suppressor cells 91. Meanwhile, CAF-rich stroma can spatially exclude cytotoxic T cells, for example through CXCL12-mediated retention at the tumor periphery 92.

The aforementioned findings underscore the immense potential of remodeling the TMME to synergize with immunotherapy. Mechanotransduction cascades centered on YAP/TAZ, Rho/ROCK, and Myosin II not only dictate the biomechanical properties of tumor cells but also profoundly modulate immune cell infiltration and resistance phenotypes through the reconfiguration of cytoskeletal architecture 93-97. Consequently, emerging "mechano-immunotherapy" strategies aim to rectify this dysregulated mechanical homeostasis via physical or chemical interventions, thereby dismantling immunosuppressive barriers. For instance, the utilization of ultrasound-driven or sonodynamic nanoplatforms to selectively degrade the ECM or induce matrix loosening has been demonstrated to significantly reduce tumor stiffness and ablate physical barriers, subsequently facilitating the deep intratumoral penetration of effector T cells and therapeutic agents 98-101. Concurrently, pharmacological interventions targeting the mechanosensitive transcription factor YAP (e.g., verteporfin) can effectively augment the efficacy of anti-PD-1 therapies, inducing a pro-inflammatory remodeling of the TME 102. In conclusion, precision targeting of the mechano-immune interface emerges as a highly promising paradigm for overcoming tumor immune resistance and improving clinical response rates.

#### 3.2.5 Mechanically Induced Chemoresistance

In late-stage tumor progression, the mechanical microenvironment evolves into a decisive factor for chemoresistance. Firstly, fibrotic ECM remodeling establishes a dense, high-pressure physical barrier. Activated CAFs drive excessive deposition and crosslinking of collagen, fibronectin and hyaluronic acid, generating a dense stromal scaffold 103. This compact matrix imposes steric constraints on drug transport, particularly for macromolecules and nanomedicines, by increasing diffusion tortuosity and producing a pronounced sieving effect 104. In addition, negatively charged ECM components such as hyaluronic acid can sequester positively charged chemotherapeutic agents, thereby reducing their bioavailable concentration within tumor tissue 105, 106.

Abnormally high IFP further strengthens this barrier 107. By diminishing the pressure gradient required for transvascular and interstitial transport, elevated IFP impedes drug penetration into the tumor core 108, 109. As a result, small-molecule agents such as doxorubicin and gemcitabine frequently accumulate in perivascular regions 110, with effective penetration often restricted to approximately 50-100 μm 111. Both experimental and clinical evidence indicate that drug delivery is markedly reduced in high-pressure tumor tissues 112, 113.

Secondly, enhanced matrix stiffness induces a chemoresistant phenotype. YAP/TAZ maintain cancer stemness in high-stiffness environments, serving as a significant reservoir for resistance 114. TAZ nuclear translocation sustains cancer stem cell (CSC) traits and enhances resistance to chemotherapy through mechanisms such as phase separation with NANOG (**Figure 3E**) 115. Moreover, matrix stiffness upregulates multidrug resistance proteins (e.g., MRP1) via the FAK-PI3K/Akt and YAP/TAZ axes, enhancing drug efflux 116. Recent research also suggests a "mechanical memory" effect where mechanical stimuli stabilize drug-resistant states at the epigenetic level, allowing cells to maintain their phenotype even after the initial stimulus is removed 117.

Thus, chemoresistance is a multi-mechanistic outcome of both physical delivery constraints and mechanical signal-driven cellular reprogramming, making the targeting of ECM stiffness a promising avenue for reversing resistance.

## 4. Modeling the Tumor Mechanical Microenvironment in Organoids

### 4.1 Organoids as Advanced Models of the Tumor Mechanical Microenvironment

Conventional 3D culture models, including tumor spheroids, scaffold-based cultures, and tumor-on-a-chip systems, have overcome some limitations of conventional 2D cultures and animal models by providing more physiologically relevant platforms to recapitulate specific features of the tumor microenvironment 118. However, these models still face substantial challenges in reproducing the hierarchical cellular architecture, dynamic mechanical microenvironment, and patient-specific heterogeneity characteristic of primary tumors. Tumor spheroids are typically generated through the aggregation of a single tumor cell line and can partially recapitulate homotypic cell-cell interactions and diffusion-driven nutrient gradients 119. However, their monocellular composition and limited structural organization result in insufficient representation of the complex tissue architecture and authentic cell-ECM interactions observed in primary tumors, thereby restricting their ability to reproduce dynamic tumor microenvironmental regulation 120. Scaffold-based 3D cultures introduce biomaterials such as collagen and hydrogels to provide ECM-like support, thereby enabling cell-matrix interactions and investigation of ECM-mediated signaling 121. Nevertheless, these systems typically rely on predefined extracellular components and simplified cellular compositions, which may not fully capture the dynamic and heterogeneous nature of the tumor microenvironment 122. Tumor-on-a-chip platforms further advance 3D tumor modeling by integrating microfluidic technologies to precisely regulate fluid flow and mechanical cues 123. However, similar to scaffold-based approaches, many tumor-on-a-chip models are established using long-term cultured tumor cell lines, which are susceptible to genetic drift, phenotypic selection, and clonal enrichment during expansion, thereby potentially limiting their capacity to faithfully represent patient-specific tumor heterogeneity 124.

Tumor organoids represent a next-generation *in vitro* cancer model. Among these, PDOs faithfully recapitulate the essential features of their parental tumors, including genetic heterogeneity, cellular hierarchical architecture, and tumor-specific phenotypic traits 17-20. Unlike conventional 3D tumor cultures that primarily depend on passive cell aggregation to generate spatial structures and diffusion gradients, PDOs actively establish complex tissue architectures through self-organization and dynamic ECM interactions, thereby enabling more physiologically relevant modeling of tumor microenvironmental regulation 20. Furthermore, by integrating biomimetic ECM hydrogels, microfluidic systems, and mechanical stimulation strategies, PDO-based platforms provide unique opportunities to reconstruct tumor mechanical features while preserving patient-specific characteristics. These models provide powerful platforms for elucidating the mechanisms by which mechanical cues within the tumor microenvironment influence tumor initiation, progression, invasion, and migration 22, 23.

### 4.2 Biomaterial-Based Models for Engineering the Tumor Mechanical Microenvironment

#### 4.2.1 Synthetic and Reconstituted Biomaterials: Engineering Matrix Stiffness in Organoid Models

Matrix stiffening during tumor progression is a key physical characteristic of TMME 125. In highly desmoplastic malignancies such as pancreatic cancer, both clinical palpation and biomechanical measurements have demonstrated that tumor tissues are substantially stiffer than their normal counterparts 126. Importantly, this increase in stiffness is not merely a pathological byproduct of disease progression; rather, it functions as an active regulatory signal that shapes tumor-cell behavior through mechanotransduction pathways 127. For these reasons, stiffness-defined organoid models provide an essential entry point for dissecting how mechanical cues influence tumor biology.

A central strategy in this area employs synthetic hydrogels with tunable mechanical properties to isolate matrix stiffness as an independent experimental variable, thereby minimizing confounding biochemical heterogeneity. In these systems, stiffness is precisely regulated by adjusting polymer concentration, crosslinking density, or network architecture, allowing investigators to modulate mechanical cues while maintaining a constant biochemical composition. For example, polymer hydrogels with defined shear storage moduli (G') of 2.6, 14.6, 22.3, and 34.0 kPa, measured by rheological testing, were used to culture colorectal cancer organoids, with the most favorable organoid growth observed in matrices with lower G' values of 2.6 and 14.6 kPa (**Figure 4A**) 128. These findings suggest that tumor growth is not simply promoted by maximal stiffening, but is instead constrained by an optimal mechanical window. Similarly, in pancreatic cancer, synthetic matrices with tunable Young's modulus (E) designed to mimic progressive tissue stiffening directly altered organoid growth states, demonstrating that rigidity alone can regulate tumor behavior even in the absence of additional stromal complexity (**Figure 4B**) 129. Such systems are particularly valuable because they reduce matrix mechanics to a tractable, quantifiable variable.

Stiffness-defined models have also revealed an important mechanistic link between matrix rigidity and drug resistance. In pancreatic cancer organoids, culture in a stiff matrix that approximates the *in vivo* tumor environment induced marked chemoresistance (**Figure 4C**) 10. This effect was associated with stiffness sensing through cell-surface receptors, which enhanced downstream programs linked to drug efflux and reduced drug susceptibility. Likewise, in a colorectal cancer model, bioprinting was used to recreate with a compressive modulus of approximately 7.5 kPa, determined by compression testing, generating a mechanically relevant environment that more accurately reflected tissue features and patient drug responses than conventional culture systems (**Figure 4D**) 130. Together, these studies indicate that stiffness is not only a determinant of growth but also a key regulator of therapeutic vulnerability.

Taken together, these studies establish stiffness-defined organoid models as the most direct approach for isolating the role of bulk matrix rigidity in tumor growth, stromal activation, and treatment resistance. As such, they serve as a conceptual and experimental foundation for more complex forms of mechanical modeling.

#### 4.2.2 Engineered Extracellular Matrices: Modeling Composition, Architecture, and Dynamic Remodeling Beyond Stiffness Alone

Stiffness-defined systems primarily isolate bulk rigidity, whereas ECM engineering seeks to reconstruct the matrix as a more physiologically relevant microenvironment. However, conventional organoid culture still relies heavily on animal-derived matrices such as Matrigel, which suffer from batch variability, poorly defined composition, and limited control over mechanical and structural properties 131, 132. These limitations make it difficult to systematically interrogate how matrix composition, topography, viscoelasticity, degradability, and remodeling collectively shape organoid behavior. Accordingly, ECM engineering has emerged as a central strategy for moving organoid models beyond generic 3D culture toward biomimetic reconstruction of the tumor matrix environment.

For example, type I collagen is particularly relevant because it is a major ECM component in solid tumors, and its abnormal deposition and remodeling are closely linked to tissue stiffening, disease progression, and poor prognosis 133. However, pure collagen hydrogels often lack sufficient mechanical robustness to reproduce the fibrotic properties of highly stiff tumors such as pancreatic cancer 134. To overcome this limitation, investigators have developed multiple strategies to modulate collagen mechanics and architecture. Physical crosslinking approaches can improve matrix stability by altering gelation temperature and fiber density 135. For example, Rezabeigi et al. demonstrated that regulation of pH during collagen gelation markedly affects the viscoelastic properties and structural organization of collagen matrices 136. Beyond single-component systems, more sophisticated approaches use multicomponent self-assembly to recapitulate the compositional complexity of native ECM. Hedegaard et al. developed peptide-protein co-assembling matrices capable of simultaneously incorporating multiple ECM features to construct organ-specific TME models (**Figure 5A**) 137. Moreover, Wang et al. developed an engineered alginate hydrogel system with independently tunable viscoelastic properties and integrin-binding capacity, demonstrating that integrin-mediated matrix sensing together with matrix viscoelasticity regulates CAFs states 138. These studies underscore that organoid morphology and function are determined not only by stiffness, but also by the biochemical composition and higher-order architecture of the matrix.

An equally important direction in ECM engineering is the introduction of dynamic and spatially organized matrix cues. Stimuli-responsive biomaterials offer a means of modeling the spatiotemporal regulation of TMEs, rather than treating the matrix as a static scaffold. Complementing tissue-level reconstruction, Devarasetty et al. demonstrated that ECM fiber alignment critically regulates colorectal cancer cell phenotype. In their colorectal liver metastasis model, LX2 cells, which are immortalized human hepatic stellate cells, actively remodeled collagen I and promoted the formation of structured collagen bundles. This organized ECM architecture promoted an epithelial-like phenotype, whereas disordered matrices induced EMT-like features and an invasive phenotype (**Figure 5B**) 139. This work illustrates that matrix engineering must account not only for bulk mechanics, but also for anisotropy, fiber organization, and cell-driven remodeling. Engineered matrix design is also essential for the generation of vascularized organoid models. Because rapid tumor expansion depends on angiogenesis, static matrices lacking vessel-like structures cannot adequately reproduce transport, signaling, or local mechanical heterogeneity. To overcome this limitation, Bray et al. developed a multi-parameter hydrogel platform incorporating pro-angiogenic factors and degradable motifs to support endothelial-network formation and tumor organoid vascularization 140.

ECM engineering is moving toward adaptive and patient-specific systems. A representative approach is patient-derived decellularized scaffolds (PDS). Blanco-Fernandez et al. used decellularized breast tissue as a bioink for printing, thereby generating a system that preserved both tissue-specific stiffness and complex biochemical cues relevant to tumor invasion 141. Additionally, Pezeshki and colleagues generated PDS from surgically resected breast tumor tissue and matched normal breast tissue (**Figure 5C**) 142. Comparative analysis revealed that tumor-derived scaffolds exhibited greater collagen density, increased glycosaminoglycan content, and a substantially higher Young's modulus than normal tissue-derived scaffolds. Functionally, tumor-derived PDS promoted MCF-7 cell proliferation, increased the expression of invasion-related genes such as CAV1, CXCR4, and MYB, and enhanced IL-6 secretion, whereas normal PDS suppressed malignant phenotypes 142. These findings indicate that tumor-specific ECM remodeling is not incidental, but is required to sustain invasive behavior. However, the broader utility of PDS is inherently constrained by the scarcity of patient-derived samples and the logistical challenges in standardizing their production 143. The availability of such scaffolds is strictly dictated by the specific clinical parameters of the donor, including tumor stage, subtype, and prior treatment history. This makes it difficult to achieve the high-throughput standardization required for large-scale drug screening 144. Consequently, while PDS provides a high-fidelity "gold standard" for mechanistic insights, current ECM engineering efforts are increasingly focusing on developing synthetic or hybrid matrices that can recapitulate patient-specific biochemical and architectural signatures without relying exclusively on inherently limited biological tissue.

Overall, ECM engineering offers a promising means of extending tumor organoid modeling beyond bulk stiffness by incorporating matrix composition, topology, fiber organization, and time-dependent remodeling. Although these approaches have not yet been widely applied to tumor organoids, their successful implementation in other 3D cancer models suggests considerable potential for reconstructing more physiologically relevant TMME within organoid systems. Thus, ECM engineering may provide an important bridge between simplified mechanical models and more physiologically instructive tumor organoid models.

### 4.3 Engineering-Based Models for Dynamic and Multiscale Mechanical Regulation

#### 4.3.1 Microfluidic Systems: Controlling Flow, Perfusion, Transport, and Microscale Confinement

Unlike stiffness-defined hydrogels and engineered ECMs, which primarily regulate the matrix context surrounding organoids, microfluidic platforms are uniquely suited to model the transport physics of the TMME. Traditional organoid culture systems based on bulk matrices such as Matrigel offer limited control over fluid flow, nutrient gradients, interstitial transport, and geometric confinement, and they are poorly adapted for standardized high-throughput analysis 21, 145. Microfluidic technologies address these limitations by enabling precise control over fluid dynamics, microscale spatial organization, and mass transport, thereby creating culture environments that more closely approximate the dynamic physical conditions of tumors *in vivo* 123.

Recent studies have demonstrated the utility of microfluidic platforms for investigating tumor cell responses to biomechanical cues under physiologically relevant conditions. Ouni et al. fabricated PEG-acrylate microcavity arrays using maskless lithography and generated substrates with tunable stiffness ranging from approximately 100-300 Pa to about 2800 Pa 145. Bladder cancer tumoroids cultured in softer matrices showed enhanced proliferation and cytoskeletal remodeling, whereas those grown in stiffer matrices activated p53 signaling and DNA damage response pathways (**Figure 6A**) 145. This study highlights how microfluidic systems can couple microscale confinement with tunable mechanics to probe context-dependent stress responses.

In addition to tunable mechanics, a major advantage of microfluidic systems is their suitability for modeling transport dynamics within the TMME 123. Zhang et al. developed a tumor-transendothelial migration-on-a-chip (TEMOC) platform using established breast cancer cell lines to model key steps of circulating tumor-cell extravasation 146. The platform integrates tumor-cell capture, endothelial barriers, and porous transport interfaces within a single system, enabling controlled analysis of tumor-vascular interactions. Computational fluid dynamics (CFD) and finite element modeling (FEM) further showed that the chip maintains a low-shear and physiologically relevant flow environment while supporting high-throughput analysis across 121 defined microenvironments (**Figure 6B**) 123, 146. In this context, the principal contribution of microfluidics is not simply miniaturization, but the ability to control flow-mediated transport and barrier interactions with high precision.

Beyond transport regulation, microfluidic fabrication has also enabled biomimetic structures that support spatially constrained tumor-cell growth. Shi et al. used coaxial microfluidic spinning to fabricate hollow methacrylate gelatin (GelMA)/polyethylene glycol diacrylate (PEGDA) microfibers, enabling control over lumen diameter and wall thickness through precise adjustment of multiphase flow rates. The resulting scaffold provided a porous 3D matrix whose elastic modulus closely resembled that of native esophageal mucosal tissue (**Figure 6C**) 147. More broadly, perfusion-enabled microfluidic platforms have supported the formation of vascularized tumor models by combining multicellular tumor spheroids with endothelial cells to generate lumenized, perfusable vascular networks. These systems enable analysis of tumor-vascular interactions and mass transport under physiologically relevant flow conditions 148. Thus, microfluidics is particularly powerful for modeling how flow, perfusion, and geometric confinement regulate tumor behavior.

A further advantage of microfluidic systems is their compatibility with automated and dynamic experimental control. Yakavets et al. developed a closed-loop microfluidic platform integrating automated fluid handling with the Bayesian optimization algorithm Gryffin 149. Using the solution containing precursors for a biomimetic hydrogel (referred to as EKGel), the system generated uniform breast cancer spheroids or patient-derived organoids in cylindrical microchannels, with each chip accommodating 1,200 organoids. Automated control of drug concentration, administration sequence, and treatment intervals enabled simultaneous testing of 12 multidrug protocols and showed that sequential dosing strategies could reduce total drug dosage by 75% while maintaining efficacy (**Figure 6D**) 149. These studies show that microfluidics enables dynamically controlled exposure and transport conditions that are difficult to reproduce in static culture systems.

In summary, microfluidic platforms contribute to tumor mechanical modeling primarily by regulating flow, perfusion, transport, and microscale confinement rather than by serving as matrix substitutes alone. Future tumor-on-a-chip systems are likely to further integrate vascular and immune components 150, incorporate dynamic mechanical stimulation such as perfusion-induced shear stress 151, and use artificial intelligence for automated experimental design and data analysis 152. These advances will continue to strengthen microfluidics as a platform for modeling the dynamic physical environment of tumors.

#### 4.3.2 Mechanical Actuation Platforms: Applying Compression, Stretch, Shear, and Acoustic Stimulation

Whereas microfluidic systems excel at controlling flow and transport, mechanical actuation platforms are designed to impose external force regimes directly onto tumor cells or organoids. These systems therefore address a distinct dimension of tumor mechanics: the active application of compression, stretch, shear, or other time-varying physical stimuli that cannot be fully encoded by matrix properties alone. As such, they provide an important complement to hydrogel- and chip-based models and have become increasingly valuable for studying how dynamic mechanical loading shapes malignant behavior.

Early work by Tse et al. provided foundational evidence that mechanical compression can drive breast cancer cells toward a more invasive phenotype 153. This observation helped establish solid stress as a biologically active force rather than a passive byproduct of tumor expansion. Subsequent studies extended this principle to other forms of force application. For example, Matsumoto et al. developed a microfluidic organoid-trapping device to immobilize human intestinal organoids and applied fluidic shear stress through controlled medium perfusion, demonstrating that fluidic stimulation could modulate organoid surface structures 154. Kalli et al. developed a compression-induced migration model using established brain cancer cell lines and demonstrated that mechanical compression promotes tumor-cell migration through activation of the MEK1/ERK1 signaling pathway, with GDF15 identified as a functional mediator of this response 155. In bone and soft tissue tumors, Marturano-Kruik et al. used a bioreactor platform to examine mechanical loading in sarcoma organoids and found that periodic stretching promoted differentiation toward a chondroid phenotype 156. Together, these studies show that externally imposed forces can regulate not only invasion and motility, but also stromal activation and lineage plasticity.

A particularly innovative extension of this concept is the use of non-contact force fields to generate scaffold-free organoids. Acoustic Virtual 3D Scaffold (AV-Scaf) technology illustrates this emerging direction. Shan et al. used acoustic radiation force and acoustic streaming generated by a 20 MHz focused ultrasound vortex field to induce cell aggregation and mechanical stimulation without requiring exogenous matrices such as Matrigel 157. Using this approach, the authors successfully constructed melanoma and breast cancer organoids in a scaffold-free format. Mechanistically, ultrasonic stimulation activated mechanosensitive ion channels, including Piezo1 and members of the TRP family, resulting in transient Ca^2+^ influx, strengthened intercellular adhesion, and enhanced calcium-dependent autocrine ECM secretion 158, 159. Functionally, when co-cultured with T cells, AV-Scaf-derived organoids induced substantially higher levels of T-cell activation than conventional Matrigel-embedded organoids, including increased frequencies of Granzyme B-positive CD8+ T cells and IFN-γ-positive cells 157. This enhanced immune accessibility was attributed to the scaffold-free architecture, which reduced physical barriers and enabled more direct immunological synapse formation between tumor cells and T cells. In parallel, magnetically actuated systems have emerged as a complementary non-contact strategy, in which magnetic nanoparticles or embedded magnetic components enable remote application of torque or localized forces under external magnetic fields. Although initially demonstrated in non-tumor organoid models such as cardiac systems, these platforms enhance tissue maturation and mechanotransduction through precisely controlled internal mechanical cues, highlighting their potential for adaptation to tumor organoids 160.

Importantly, active mechanical stimulation may affect cells not only through direct mechanosensing but also through dynamic remodeling of the surrounding matrix or cell-matrix interface. Mechanical compression, for example, has been associated with increased fibronectin deposition and strengthened cell-matrix adhesion 153. In matrix-containing systems, external loading may further induce matrix compaction, fiber reorganization, or strain-dependent stiffening, thereby altering force transmission to embedded cells 156. Thus, cellular responses to active mechanical stimulation may reflect both direct mechanosensing and secondary effects of load-induced matrix remodeling. Accordingly, interpretation of cellular phenotypes under active actuation should consider both the applied mechanical input and the dynamic evolution of the surrounding matrix microenvironment.

Overall, mechanical actuation platforms expand tumor organoid modeling from passive reconstruction of tissue properties to active force application. They are therefore particularly useful for interrogating how dynamic and externally imposed mechanical stresses influence tumor invasion, stromal reprogramming, and tumor-immune interactions.

#### 4.3.3 Multicellular Reconstructive Models: Capturing Cell-Driven Mechanical Remodeling within the Tumor Ecosystem

Mechanical properties in tumors are not determined solely by materials or externally applied forces; they are also continuously generated and remodeled by the resident cellular ecosystem. *In vivo*, tumor cells coexist with fibroblasts, immune cells, endothelial cells, and other stromal populations that collectively reshape ECM architecture, generate traction forces, and modulate tissue tension. Multicellular co-culture models therefore address a distinct but essential dimension of tumor mechanical modeling: they reconstruct the cell-driven processes that produce and maintain the mechanical microenvironment 161.

Among stromal populations, CAFs are particularly important because they regulate both ECM mechanics and immunosuppressive signaling. Recent spatial transcriptomic studies have revealed pronounced heterogeneity in the spatial distribution and functional states of CAFs, implying that these cells contribute directly to the formation of intratumoral mechanical gradients and to the migration trajectories of immune cells 162. Consistent with this concept, co-culture systems that combine tumor organoids with fibroblasts have been shown to enhance ECM deposition, promote EMT, and increase tumor invasiveness 163. These findings indicate that stromal cells modulate tumor behavior through tightly coupled biochemical and mechanical mechanisms. Mesenchymal stromal cells (MSCs) can play related roles. Recent work has shown that MSCs facilitate organoid assembly by secreting growth factors and regulating integrin signaling, while also helping to maintain intercellular mechanical coupling within 3D structures 164. Thus, the inclusion of stromal cells in organoid models is not only a matter of cellular complexity, but also a means of reconstituting the mechanical labor that shapes tumor architecture.

Immune cells introduce an additional dimension of reciprocal mechanical regulation. Tumor-infiltrating T cells and macrophages do not merely respond to matrix conditions; they also participate in ECM remodeling through contractile and migratory forces 165. Conversely, matrix mechanics feedback on immune-cell behavior. Harder matrix environments have been associated with reduced T-cell proliferation and cytotoxicity, a phenomenon that may contribute to the immune-privileged nature of many tumors 77. On this basis, organoid co-culture systems incorporating autologous T cells, tumor-infiltrating lymphocytes, or natural killer cells have gained importance as models that capture both immune function and its mechanical context 166. Their value in this review lies less in immunotherapy prediction per se than in demonstrating that tumor immunity is inseparable from the physical microenvironment in which immune cells migrate, engage, and kill.

More broadly, multicellular co-culture moves tumor organoids beyond monocellular systems by reconstructing reciprocal interactions among matrix-producing, force-generating, and force-sensing cell populations. In this sense, multicellular co-culture is best understood as a reconstructive approach for modeling cell-mediated mechanical remodeling, rather than simply as an additive strategy for increasing biological complexity.

#### 4.3.4 3D Bioprinting Strategies: Programming Spatially Heterogeneous Mechanics and Tissue Organization

Compared with other strategies, 3D bioprinting provides the distinct capability of spatially programming cells, biomaterials, and mechanical properties. In conventional tumor organoid culture, tissue architecture, stiffness gradients, and intercellular mechanical relationships emerge in a relatively uncontrolled manner. By contrast, 3D bioprinting enables the precise deposition of cells, bioinks, and growth factors into predefined geometries, allowing investigators to fabricate tumor constructs with programmable structure and mechanics 167. This makes bioprinting particularly valuable for modeling regional heterogeneity within tumors.

A major contribution of 3D bioprinting is its ability to recreate structural heterogeneity in a controlled and reproducible fashion. By spatially arranging different cell populations and matrix materials, bioprinting can generate tumor constructs that mimic compartmental variation in cellular composition, local stiffness, and microenvironmental context. Recent studies have shown that such strategies can reconstruct intratumoral heterogeneity and reproduce differential drug responses across spatially distinct niches (**Figure 7A**) 168. Advanced bioprinting approaches have further enabled the accurate positioning of multiple cell populations and bioinks, thereby facilitating the construction of tumor organoids with biomimetic architecture and microenvironmental heterogeneity 169. In glioma, integration of 3D bioprinting with computational analysis has produced models with controllable microenvironmental features, offering insights into how heterogeneity shapes therapeutic response 170. These examples illustrate that the unique value of bioprinting lies in its capacity to encode heterogeneity directly into the model rather than allowing it to emerge stochastically.

Another important application of bioprinting is the generation of mechanically coupled multicellular systems. By printing tumor cells together with stromal and immune cells, it becomes possible to define interaction interfaces, stiffness zones, and force-transmission pathways with much greater precision than in conventional self-organizing culture. For example, a 3D-bioprinted breast cancer model containing tumor cells and CAFs demonstrated that stromal remodeling increased matrix stiffness and altered tumor-cell behavior and radiosensitivity through combined paracrine and mechanical coupling mechanisms (**Figure 7B**) 171. Likewise, biomimetic 3D tumor constructs integrating tumor, stromal, and immune cells have successfully reconstructed functional tumor immune microenvironments in which multicellular crosstalk promoted drug resistance and more physiologically relevant tumor phenotypes (**Figure 7C**) 172. In this respect, bioprinting does not simply add more cell types; it organizes them spatially so that local mechanics and intercellular interactions can be more deliberately controlled.

Recent advances have also improved the reproducibility and scalability of bioprinted tumor models. Machine learning-assisted frameworks have been developed to optimize print fidelity, extrusion parameters, and structural stability 173. For instance, Ruberu et al. established a machine learning-guided strategy capable of predicting print outcomes and improving the reproducibility of hydrogel-based constructs by optimizing variables such as pressure and speed (**Figure 7D**) 174. Related studies have shown that deep learning can quantitatively connect printing parameters with structural outcomes, enabling increasingly data-driven control of construct formation 175. At the same time, automated and high-throughput bioprinting workflows are making it possible to fabricate larger numbers of standardized tumor organoid models, which is valuable for quantitative studies of mechanically regulated phenotypes and for drug-testing applications 176.

Taken together, 3D bioprinting occupies a distinct methodological niche in tumor mechanical modeling by enabling programmable spatial heterogeneity, controlled multicellular arrangement, and reproducible fabrication. It is therefore particularly well suited for building organoid models in which local mechanics and tissue architecture must be specified rather than merely approximated.

To provide a comprehensive and clear comparison of the various tumor mechanical environment modeling strategies discussed above, they are systematically summarized in Table 1.

## 5. Limitations and Perspectives

In this review, we have summarized current strategies for biomimetically modeling the TMME in organoid systems and highlighted their applications in mechanistic studies and therapeutic evaluation. The rapid development of organoid technology has provided an important experimental platform for investigating tumor initiation, progression, and treatment response 177. Organoids are particularly well suited for tumor mechanobiology because they function as self-organizing, feedback-regulated mechanical systems that recapitulate endogenous force generation, spatial mechanical heterogeneity, and long-term biomechanical evolution 178. In contrast, engineered bioprinted constructs and microfluidic tumor-on-a-chip platforms primarily impose externally defined mechanical constraints 179. Nevertheless, despite substantial progress, current organoid-based models still face important limitations in their ability to faithfully reconstruct the mechanical dimension of the TME. These limitations constrain both mechanistic interpretation and the broader clinical translation of organoid platforms.

The first major limitation of many current tumor organoid models is their predominant focus on matrix stiffness as a single mechanical variable 180. Other factors such as shear stress, compression, matrix tension, and topography remain insufficiently explored, limiting the physiological relevance and biomimetic fidelity of these systems 25, 181. Beyond the incomplete representation of individual forces, the more crucial challenge lies in achieving the biomimetic reconstruction of a coupled mechanical microenvironment within the tumor organoid model. In solid tumors, cell-generated traction, ECM mechanics, interstitial fluid flow, and solid stress are closely interconnected 182. Therefore, reproducing this complex environment in organoid systems through the regulation of a single mechanical parameter remains inherently limited.

At the same time, the simultaneous modulation of multiple mechanical cues within tumor models introduces a key interpretational challenge, as their coupling obscures causal attribution and complicates the prioritization of physiologically relevant design parameters. To address this challenge, future efforts should focus on developing multi-parameter, independently controllable organoid models, combined with computational modeling and quantitative imaging, to dissect the individual and synergistic effects of distinct physical cues.

A second major bottleneck is the limited reproducibility and standardization of current organoid culture systems. Recent studies have emphasized that organoid models often show substantial variability across laboratories, batches, and even nominally identical experimental conditions 131. This variability arises from intrinsic biological heterogeneity, differences in the microenvironment, and inconsistencies in experimental procedures. Importantly, differences in matrix formulation, mechanical characterization methods, culture conditions, and biological sources can produce inconsistent mechanical and biological responses across studies, making apparently conflicting findings difficult to distinguish from platform-dependent effects. Because organoids rely on self-organization, they are highly sensitive to changes in matrix composition, nutrient and oxygen availability, and cell-cell signaling. Thus, poor control of the microenvironment and a lack of standardized protocols across laboratories remain major barriers to reproducibility 183. Beyond technical variability in culture procedures, standardized quality assessment and validation of organoid models also remain insufficient. No widely accepted framework has been established to assess organoid maturity, function, and similarity to the corresponding *in vivo* tissues. Current assessments include morphological analysis, immunofluorescence staining, and mechanical parameter measurements; however, differences in assessment criteria and measurement methods can limit reproducibility and comparability across laboratories 180, 183. Moreover, direct comparison of organoid cells with their corresponding source tissues is important for assessing cellular fidelity and improving the reliability and comparability of organoid models 184. Cross-platform benchmarking against common reference materials, standardized mechanical assays, and matched source tissues will therefore be important for determining whether measurements obtained using different organoid platforms and laboratories are quantitatively comparable. Mechanical parameters should also be reported using standardized physical quantities and SI units wherever applicable, for example, Pa or kPa for elastic moduli and viscoelastic parameters such as storage and loss moduli, and Pa for shear or compressive stress, together with clearly defined testing conditions and analysis procedures to facilitate cross-study comparison. Establishing standardized and quantitatively controllable culture and evaluation systems, including calibrated mechanical parameters, stable matrix properties, and objective validation criteria, is therefore essential for improving the reproducibility, comparability, and translational reliability of organoid models.

A third limitation is the limited ability of current tumor organoid systems to reproduce the spatial heterogeneity and tissue architecture of the TMME. Although organoids better capture tumor heterogeneity than conventional 2D systems 185, their surrounding ECM often lacks the complex fiber organization found *in vivo*, including heterogeneous collagen architectures, variable fiber diameters and orientations, and dynamic remodeling 186. Most hydrogel-based systems form relatively homogeneous networks and therefore cannot fully reproduce patient-specific ECM topologies or spatially organized mechanical cues 187. Although ECM-derived hydrogels partially recapitulate native ECM properties, they still provide limited control over multiscale architecture and dynamic remodeling. Moreover, mechanical regulation is often overlooked in culture protocols, which may impose selective pressure during long-term passaging and lead to the loss of mechanically relevant subpopulations and patient-specific cellular and mechanical phenotypes. Such phenotypic drift may reduce organoid fidelity. Future organoid platforms should therefore better integrate spatially organized biochemical and mechanical cues while minimizing culture-induced phenotypic drift.

Taking together, these limitations highlight the challenges that remain for tumor organoid models to achieve broader biological and clinical utility. Future advances in tumor organoid mechanobiology will require standardized and highly biomimetic systems that integrate multiple mechanical factors while preserving relevant biological phenotypes and enabling clinically meaningful validation. Beyond improving model fidelity, translation into drug-development workflows will require standardized qualification criteria, validated assay performance, defined quality-control metrics, and regulatory frameworks that account for variability in biological source materials, matrices, culture procedures, and mechanical measurements. The goal is therefore not simply to maintain tumor growth in 3D, but to develop experimentally controllable systems in which relevant physical parameters can be defined, perturbed, quantitatively measured, and linked to biologically and clinically meaningful outcomes.

One important direction toward this goal is the development of engineered ECM materials with greater structural precision and tunability. Such materials offer an opportunity to move beyond Matrigel while better reproducing the mechanical and biochemical features of native tissues. In recent years, polyisocyanopeptide (PIC) derivatives, self-assembling peptides, and programmable peptide-based materials have been used to construct 3D networks with controllable secondary structures and tunable mechanical properties. These systems can be molecularly designed to stabilize β-sheet conformations and generate nanoscale fibrillar bundle architectures. For example, the self-assembling peptides developed by Pang et al. spontaneously form nanofiber networks under physiological conditions, producing hydrogels with tunable mechanics and good cytocompatibility 188. Similarly, Haritha Asokan-Sheeja et al. reported the *in situ* synthesis and self-assembly of peptide-PEG conjugates to create a double-network hydrogel incorporating β-sheet secondary structures, yielding a stable nanofibrous matrix with controllable mechanical and biological properties 189. Compared with Matrigel, such engineered materials offer superior batch consistency and greater design flexibility. Their future integration with PDOs may enable more accurate matching of individualized mechanical parameters.

To achieve high-fidelity biomimetic simulation of the mechanical microenvironment in tumor organoids, interdisciplinary technical integration is still required. The construction of standardized organoid platforms increasingly depends on the convergence of bioengineering, materials science, microfluidics, automation, and artificial intelligence. Recent studies have shown that microfluidic control combined with real-time monitoring can help maintain standardized culture conditions while reducing variability introduced by manual operation 190. More broadly, automated systems integrating sensors, sampling modules, and longitudinal monitoring can generate quantitative data throughout organoid growth and thereby support high-throughput analysis and model standardization 191. Microfluidic chips can also be used to apply well-defined mechanical inputs, including fluid shear stress, cyclic compression, and biochemical gradients, while real-time imaging and AI-assisted analysis may provide objective and quantitative evaluation criteria 192. It helps to maintain a stable and controllable physical environment during organoid cultivation, thereby facilitating the precise reconstruction of the mechanical microenvironment. In parallel, high-resolution 3D microscopy can quantify features such as fiber orientation and pore-size distribution in patient tissues, and machine-learning algorithms can translate these parameters into manufacturable design specifications for biomimetic matrices 193. Standardized data-acquisition and parameter-sharing platforms will be essential for enabling cross-laboratory comparison and improving reproducibility at the field level 194.

By developing and integrating the aforementioned technologies, it is possible to create a tumor tissue model that can personalize the reconstruction of the patient's specific mechanical microenvironment. By integrating patient-derived organoids with customized ECM mechanical properties, such models can better capture interpatient heterogeneity and improve the prediction of individual responses to radiotherapy, chemotherapy, targeted therapy, and immunotherapy 195. Moreover, tailoring mechanical features to reflect those of distinct metastatic niches may provide deeper insight into organ-specific metastasis and therapy resistance. Collectively, these advances are expected to facilitate patient-specific drug sensitivity testing and support individualized treatment strategies, advancing organoid platforms toward clinically actionable precision medicine.

In summary, the future of tumor organoid research lies in building experimentally tractable yet physiologically faithful systems in which multiple mechanical cues, matrix architectures, stromal interactions, and spatial gradients can be integrated in a standardized manner. Achieving this goal will require not only improved biomaterials and engineering platforms, but also a conceptual shift from single-parameter modeling toward systems-level reconstruction of the TMME. Such efforts are likely to define the next generation of organoid models for both mechanistic discovery and translational cancer research (**Figure 8**).

## 6. Conclusion

In summary, the TMME plays a pivotal role in cancer initiation, progression, and therapeutic response, complementing traditional genetic and biochemical perspectives. Mechanical cues, including matrix stiffness, solid stress, and interstitial fluid flow, critically regulate tumor cell behavior and disease evolution. However, conventional models fail to accurately recapitulate these dynamic and complex biomechanical features, limiting their translational relevance. Emerging NAMs are reshaping cancer research by promoting human-relevant, animal-free experimental systems.

By preserving selected features of tumor heterogeneity and 3D organization, PDOs provide a useful foundation for modeling tumor mechanics. Integration with engineered biomaterials, microfluidic systems, mechanical stimulation, bioprinting, and multicellular co-culture enables controlled reconstruction of key mechanical features, including matrix mechanics, fluid flow, compressive forces, spatial organization, and ECM remodeling. However, the extent of biological validation varies among these approaches, with some remaining at the proof-of-concept or early-stage level. These advances provide new opportunities to investigate how mechanical cues regulate tumor development, invasion, and therapeutic responses and to develop more physiologically relevant organoid models.

Despite these advances, current organoid models remain limited in recapitulating TMME. Most studies focus on matrix stiffness, while neglecting the dynamic and coupled nature of multiple mechanical cues. In addition, limited reproducibility due to poorly defined biomaterials and non-standardized culture conditions, together with insufficient representation of ECM architecture and microenvironmental gradients, constrains physiological relevance. Addressing these challenges will require more standardized and integrative strategies through the convergence of advanced biofabrication, biomaterials, and multi-omics technologies. Such progress is expected to enable more biomimetic tumor organoid models that better preserve patient-specific tumor characteristics and deepen our understanding of tumor mechanobiology.

## Figures and Tables

**Figure 1:**
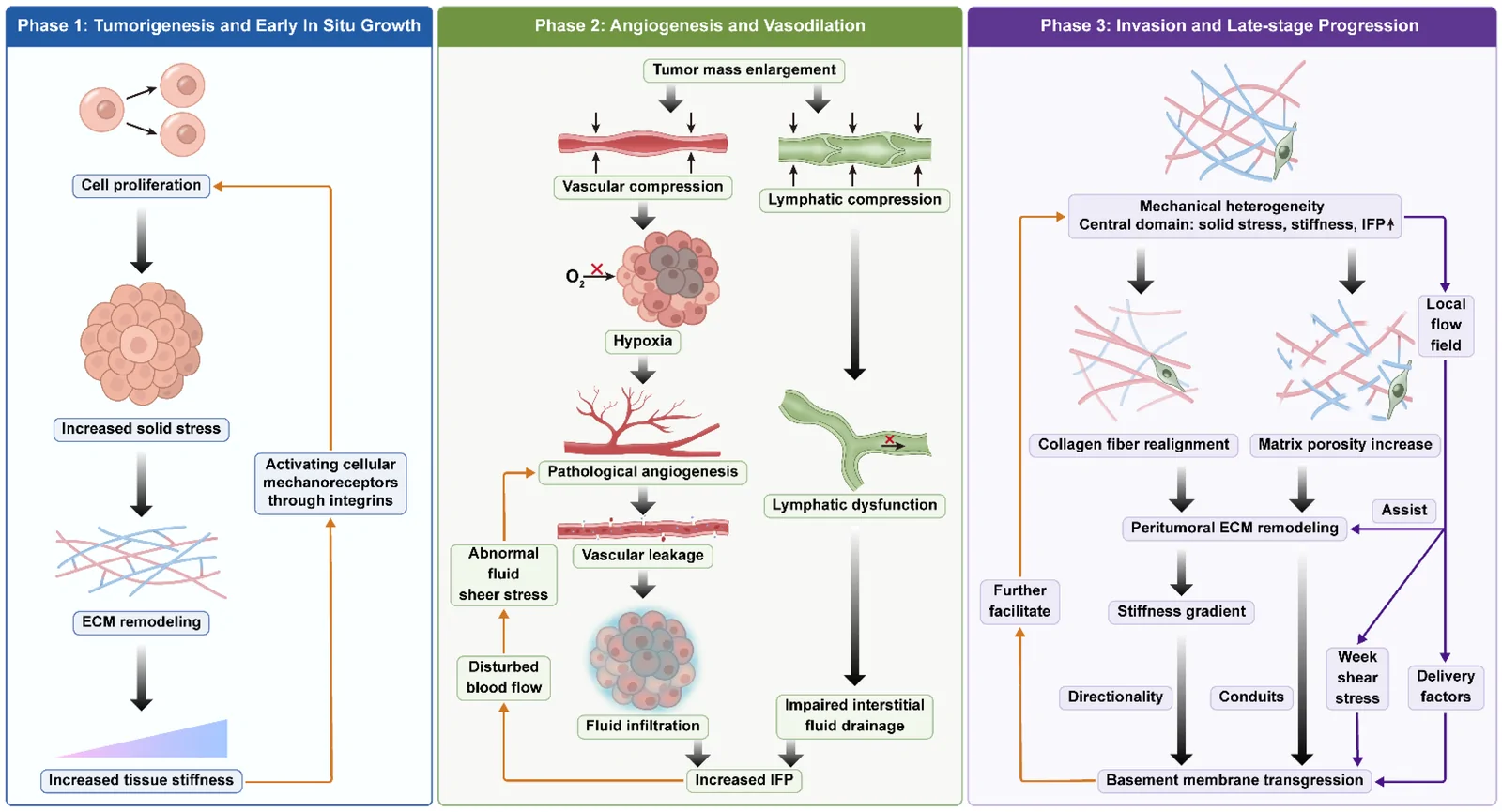
Schematic illustration of the mechanical microenvironment evolution across different stages of tumor progression. Created with Procreate and Adobe Illustrator.

**Figure 2:**
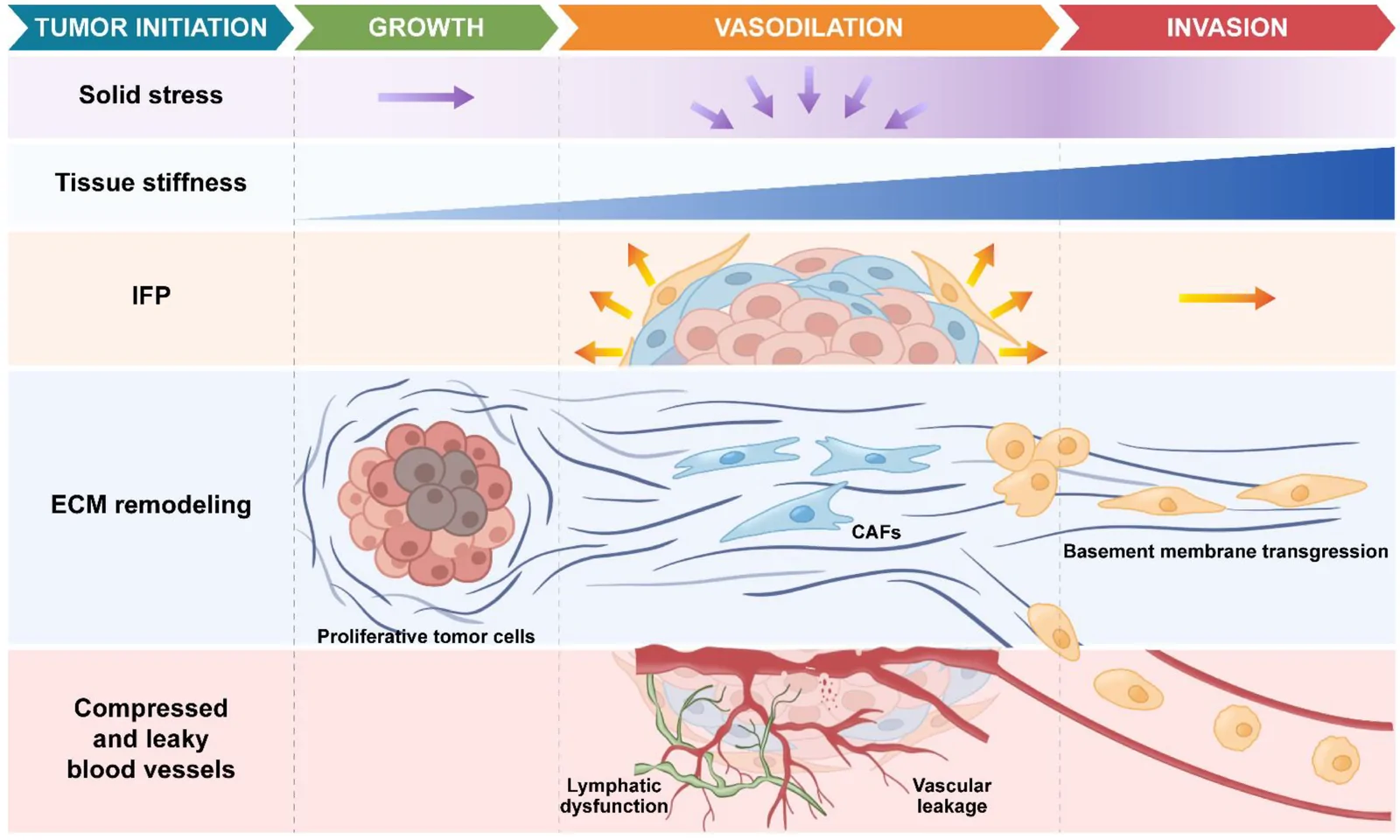
Parallel evolution of biomechanical parameters and structural hallmarks across the longitudinal progression of tumors. Created with Procreate and Adobe Illustrator.

**Figure 3:**
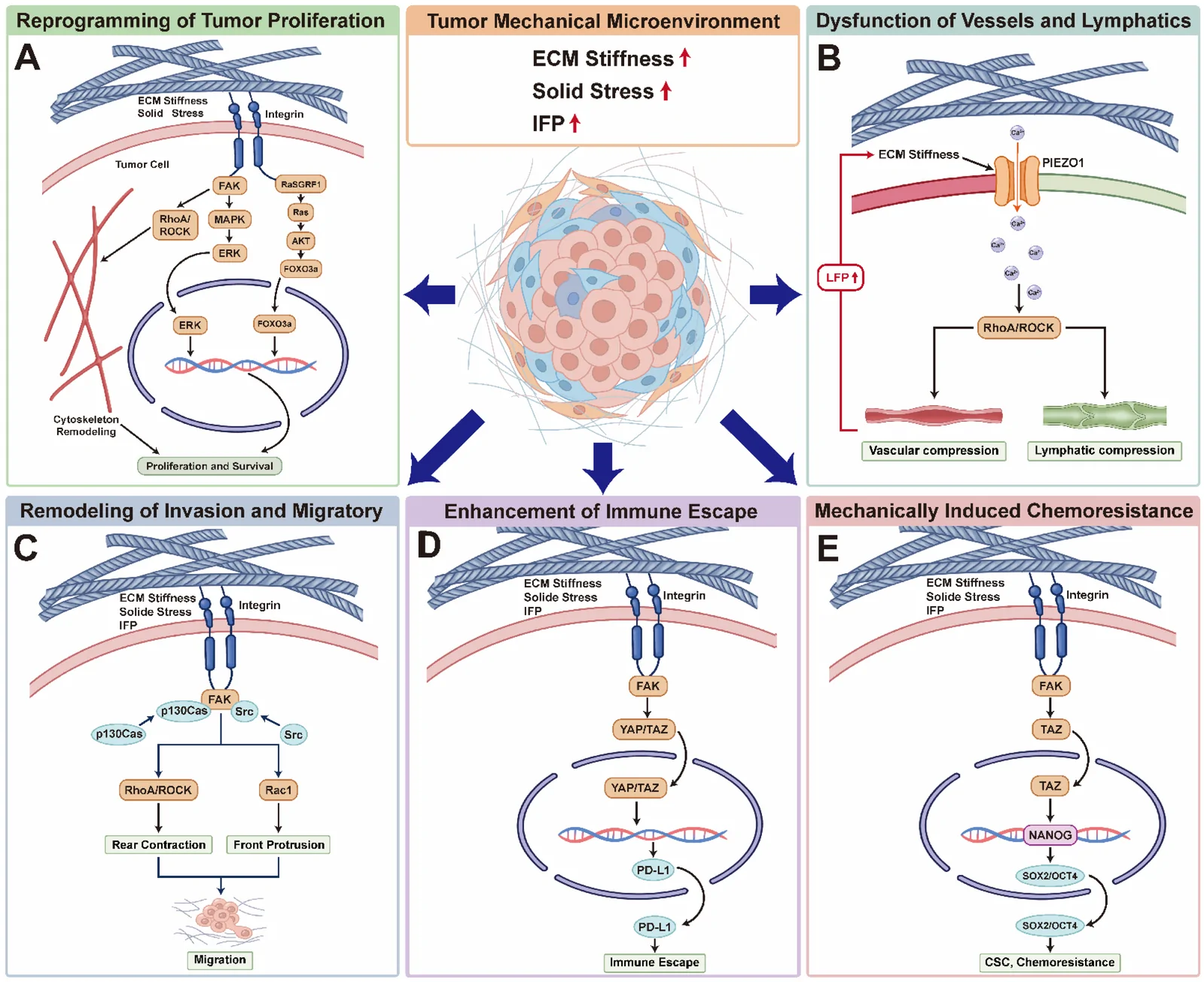
Representative mechanotransduction pathways by which the TMME drives cancer progression. (A) ECM stiffening promotes integrin clustering and activation of the FAK/Src-RhoA/ROCK-YAP/TAZ axis, enhancing tumor cell proliferation and survival. (B) Solid stress and mechanical compression activate mechanosensitive ion channels such as PIEZO1, inducing Ca²⁺ influx and downstream cytoskeletal remodeling. (C) Mechanical cues activate integrin-FAK/Src signaling to coordinate Rac1-mediated and RhoA/ROCK-dependent migration. (D) Mechanical signaling further promotes immune evasion through YAP/TAZ-dependent PD-L1 upregulation. (E) The enhanced matrix hardness induces chemotherapy resistance through the FAK-TAZ pathway. Created with Procreate and Adobe Illustrator.

**Figure 4:**
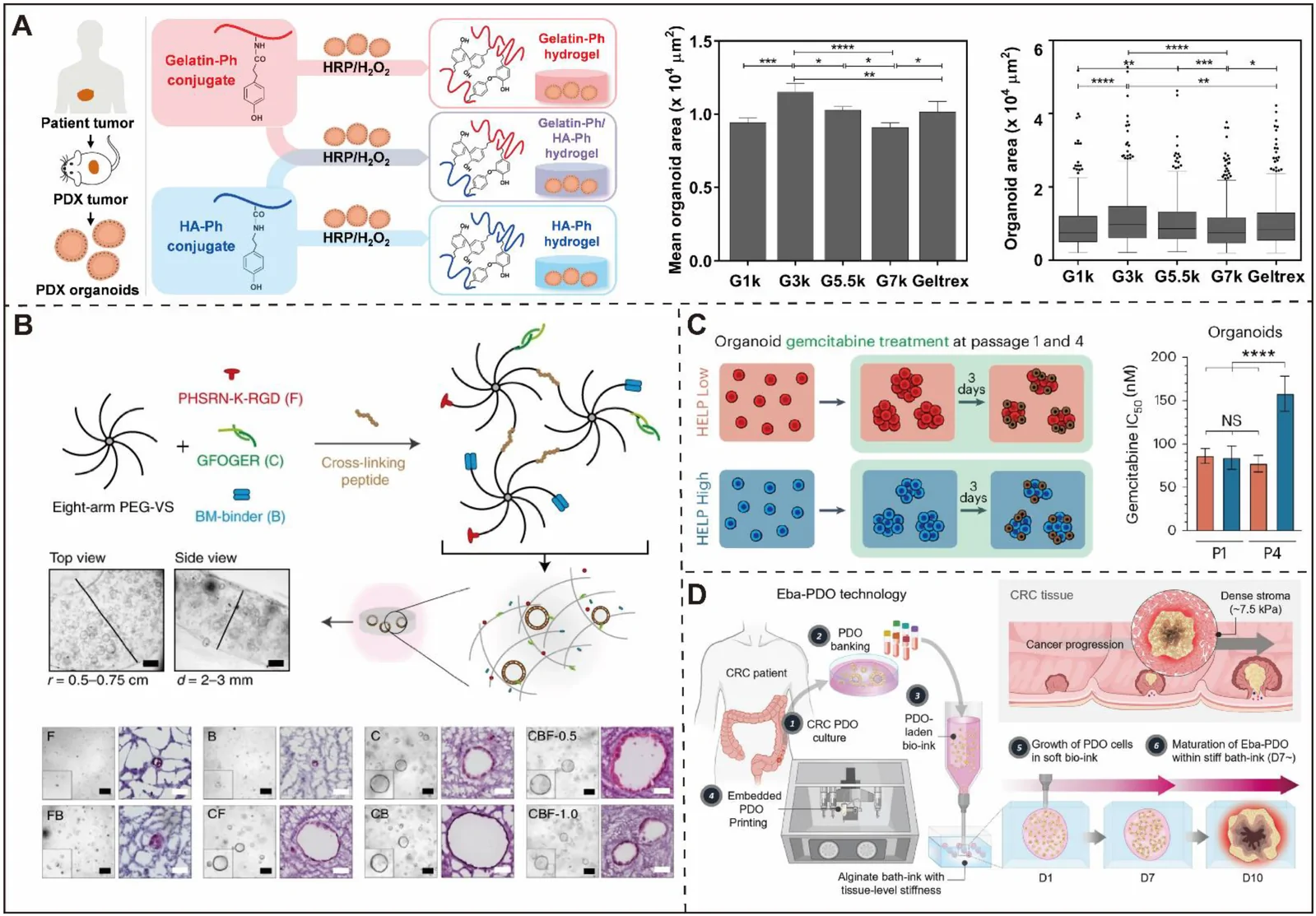
Engineered hydrogels with tunable stiffness modulate tumor organoid growth and drug response. **(A)** PDX tumor-derived organoid encapsulation in enzymatically crosslinked HA-Ph, gelatin-Ph or gelatin-Ph/HA-Ph composite hydrogels. Colorectal cancer (CRC)-PDX organoids encapsulated in mechanically defined gelatin-Ph hydrogels (5% w/v) exhibited maximal growth in moderately stiff G3k hydrogels. **(B)** 3D Polyethylene Glycol (PEG) hydrogel scaffold crafting and organoid encapsulation. Representative brightfield and H&E images of murine pancreatic cancer organoids (mPCOs) in 3D PEG hydrogels using adhesion-mimetic peptides as indicated (d4, n = 3). Brightfield scale bar, 200 µm; H&E scale bar, 50 µm. **(C)** Pancreatic ductal adenocarcinoma (PDAC) organoids are treated with gemcitabine for 3 days following the formation of ~75-μm-diameter organoids, and the IC50 value of gemcitabine was calculated. **(D)** A schematic illustrating embedded 3D bioprinting process of PDO-ink based on a Geltrex™ hydrogel within an alginate bath, designed to mimic the natural CRC tissue surrounded by a rigid matrix, highlighting its importance in cancer progression. **(A)** Reproduced by permission from Elsevier, Biomaterials 128, Copyright © 2019 Elsevier Ltd. **(B)** Reproduced by permission from Springer Nature, Nature Materials 129, Copyright © 2022 The Authors, under exclusive license to Springer Nature Limited. **(C)** Reproduced by permission from Springer Nature, Nature Materials 10, Copyright © 2024 The Authors, under exclusive license to Springer Nature Limited. **(D)** Reproduced by permission from Advanced Science 130, Copyright © 2025, The Author(s), under the CC BY 4.0 license.

**Figure 5:**
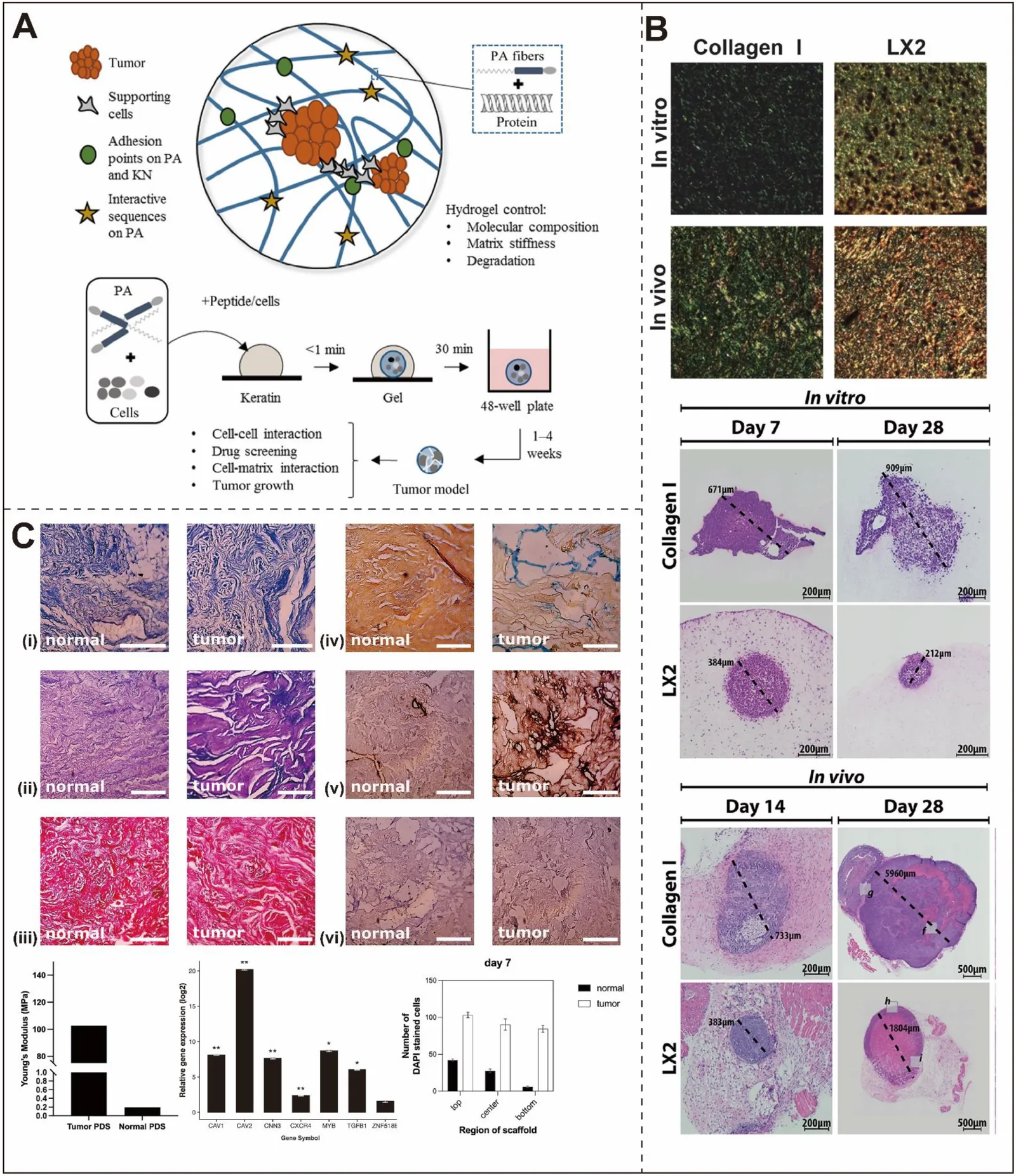
Matrix composition and structural dynamics regulate tumor organoid phenotype and function. **(A)** Schematic of a peptide-protein co-assembled hydrogel and cell encapsulation within the matrix. **(B)** 3D colorectal tumor-stroma co-culture models *in vitro* and *in vivo*. Fiber bundling was quantified by signal hue analysis, and tumor spheroid morphology was assessed on days 7 and 28 in collagen-only and LX2 co-culture constructs. **(C)** Histological and immunohistochemical analyses of normal and tumor-derived PDS showing increased collagen/glycoprotein accumulation, higher collagen IV expression, and greater stiffness in tumor PDS. Absence of CK7 confirmed complete decellularization. Tumor PDS also promoted higher expression of invasion-related genes and greater MCF-7 cell accumulation than normal PDS. Scale bar: 100 μm. **(A)** Reproduced by permission from Science Advances 137, Copyright © 2020, The Authors, under the CC BY-NC 4.0 license. **(B)** Reproduced by permission from Scientific Reports 139, Copyright © 2020 The Authors, under the CC BY 4.0 license. **(C)** Reproduced by permission from Springer Nature, Scientific Reports 142, Copyright © 2025 The Authors, under the CC BY-NC-ND 4.0 license.

**Figure 6:**
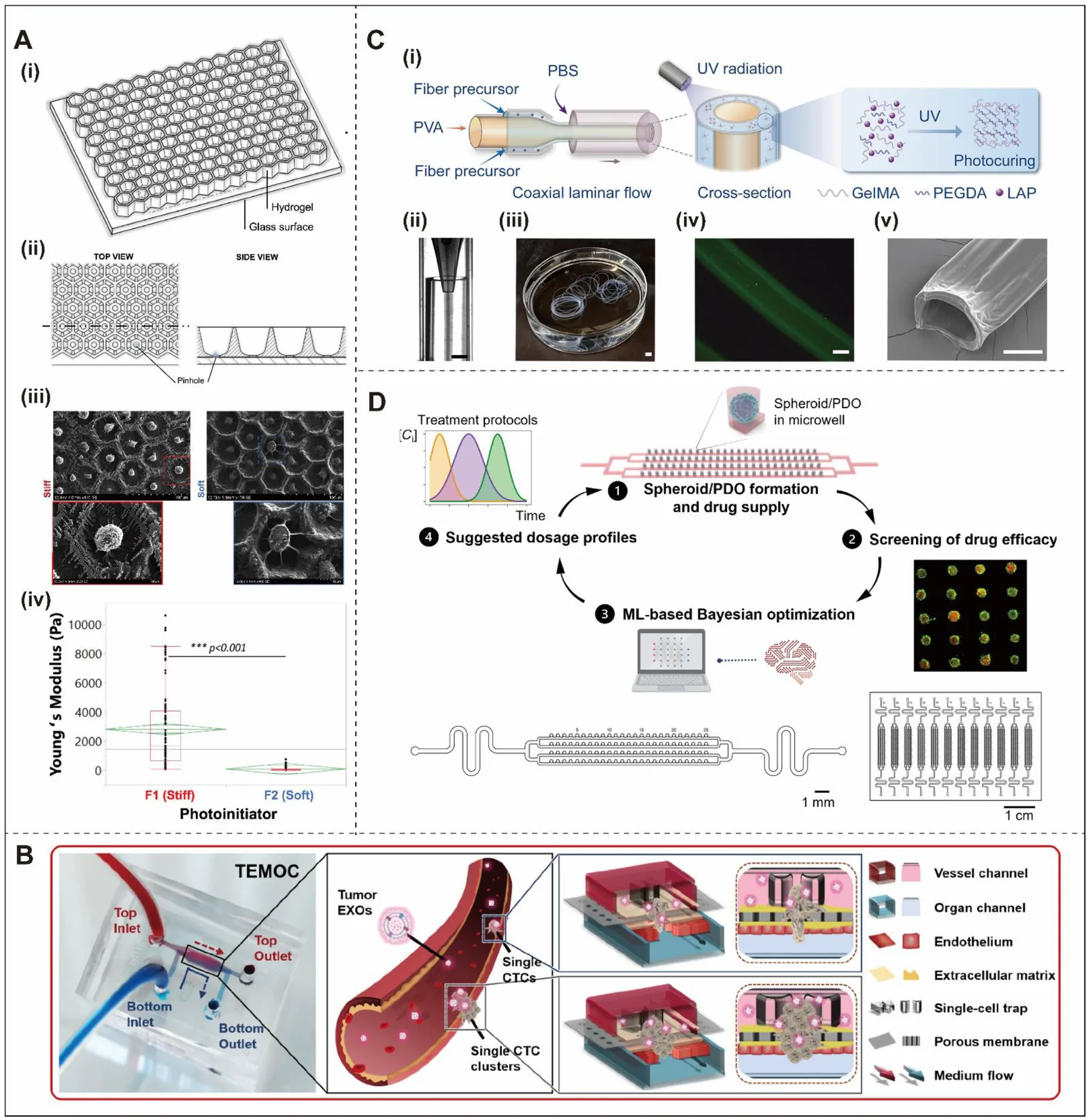
Microfluidic platforms enable precise control of tumor organoid architecture and mechanical microenvironment. **(A)** Microcavity array design, tumoroid-matrix interactions, and stiffness characterization of hydrogels with different Young's moduli. **(B)** TEMOC platform for investigating the effects of EMT phenotype on low-metastatic breast cancer cells. **(C)** Microfluidic spinning process and characterization of hollow GelMA/PEGDA microfibers. **(D)** Schematic representation of a closed-loop workflow for drug screening based on a quadruplet unit design.** (A)** Reproduced by permission from Elsevier 145, Copyright © 2025 The Authors. Published by Elsevier Inc. **(B)** Reproduced by permission from American Chemical Society, ACS Nano 146, Copyright © 2025 American Chemical Society. **(C)** Reproduced by permission from Elsevier, Cell Reports Physical Science 147, Copyright © 2025 The Authors. **(D)** Reproduced by permission from Science Advances 149, Copyright © 2025, The Authors, under the CC BY 4.0 license.

**Figure 7:**
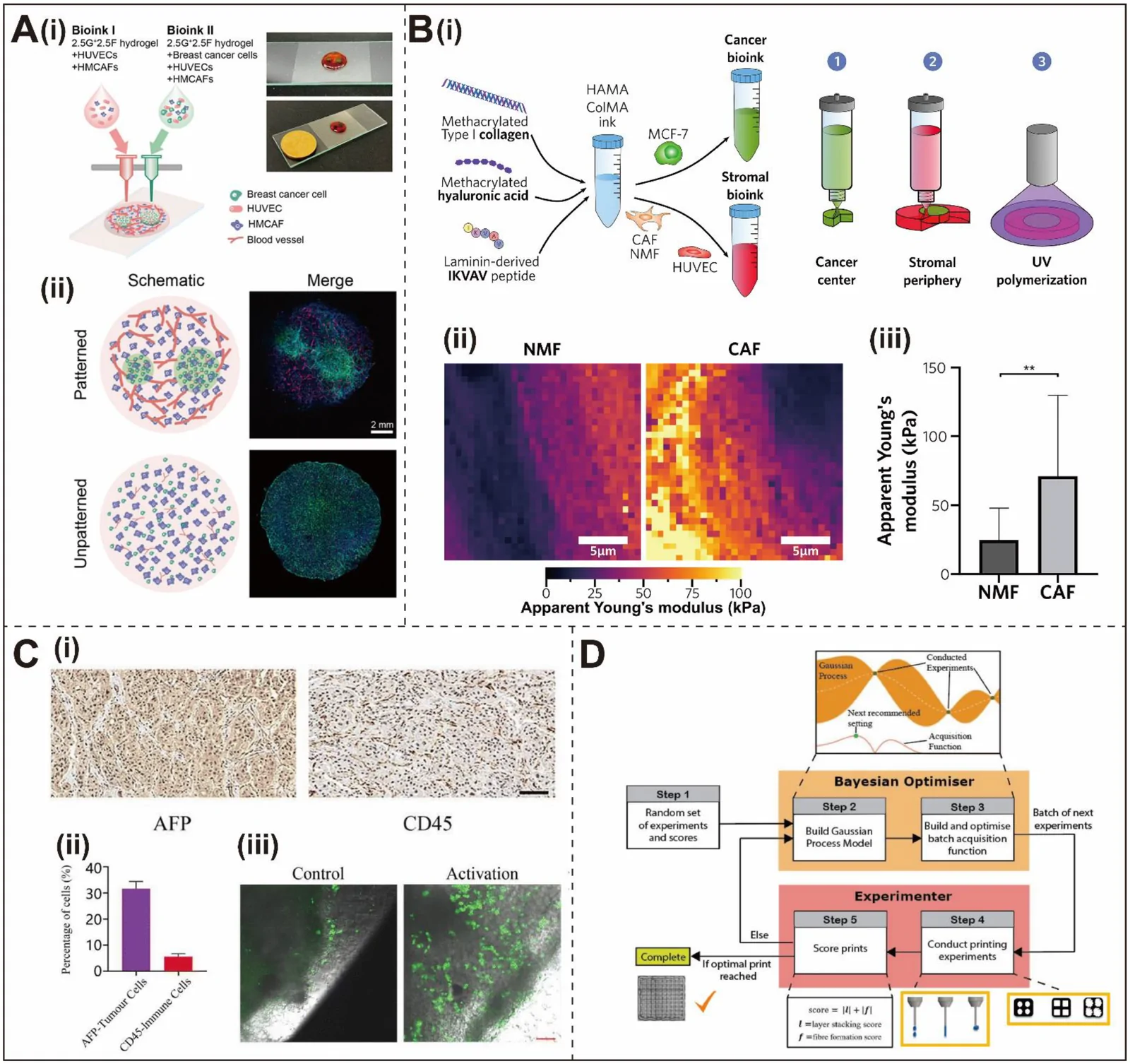
3D bioprinting reconstructs heterogeneous TME through spatial control and multicellular interactions.** (A)** Schematic of multi-nozzle extrusion bioprinting for breast tumor models using two bioinks, generating distinct cancer cell-rich (CCR, green) and stroma-rich (SR, red) regions. **(B)** Microextrusion-bioprinted breast cancer models with separate tumor and stromal compartments. AFM analysis revealed stromal stiffness differences between NMF and CAF regions. Scale bar, 5 μm. **P < 0.01. **(C)** Immunohistochemical staining of AFP and CD45 in bioprinted liver tumor models with AFP:CD45 quantification (6:1). Co-culture of HepG2, TEC, and CAF within decellularized extracellular matrix scaffolds (dLECMs) generated activated tumor microenvironments. Scale bars, 100-200 μm. **(D)** Bayesian optimization-guided closed-loop framework for iterative optimization of 3D bioprinting parameters. **(A)** Reproduced by permission from Elsevier, Trends in Biotechnology 168, Copyright © 2024 Elsevier Ltd. **(B)** Reproduced by permission from Elsevier, Bioactive Materials 171, Copyright © 2024 The Authors, under the CC BY-NC-ND 4.0 license. **(C)** Reproduced by permission from The Royal Society of Chemistry, Biomaterial Science 172, Copyright © 2024 The Royal Society of Chemistry. **(D)** Reproduced by permission from Elsevier, Applied Materials Today 174, Copyright © 2021. Elsevier Ltd.

**Figure 8:**
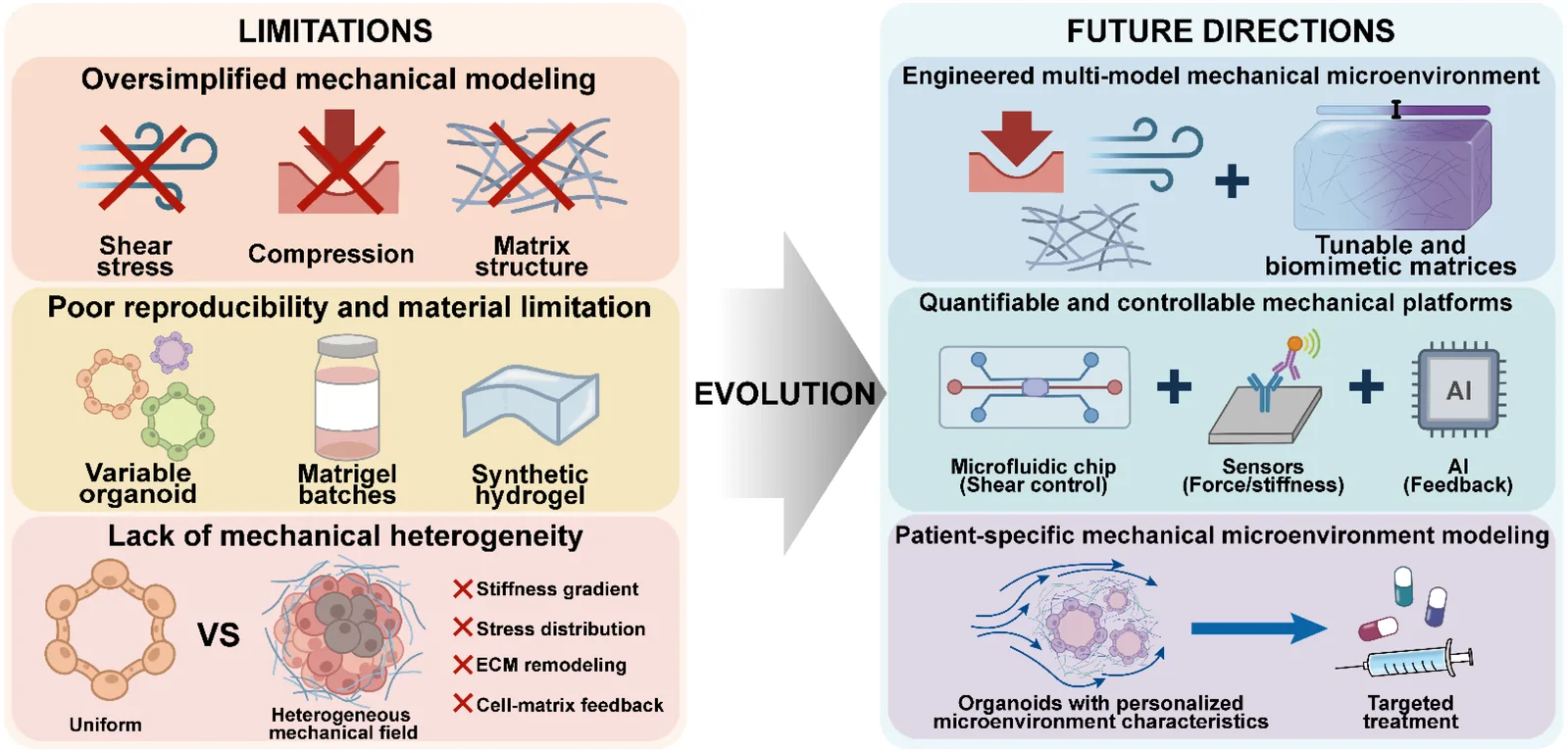
Limitations and future directions of biomimetic mechanical microenvironment modeling in tumor organoids. Created with Procreate and Adobe Illustrator.

**Table 1:** Comparison of major strategies for modeling the TMME of tumor organoids

Modeling strategy	Construction principle	Mechanical characterization methods	Representative technology	Main advantage	Validation stage	Translational potential	Ref.
Synthetic and reconstructed biomaterials	The matrix stiffness was separated and investigated as a single variable.	Rheological and compressive mechanical testing	Polymer hydrogels with a specific stiffness.	The matrix mechanics is simplified into quantifiable variables, facilitating the separate analysis of the independent effect of stiffness on tumors.	Biologically and mechanistically validated	Patient-specific drug testing	10 130
Engineered ECM	Reconstructing the matrix components, topological structure and fibrous organization.	Rheological and viscoelastic testing; compression testing	Peptide-protein co-assembling matrices; Patient-derived decellularized scaffolds (PDS).	Overcoming the limitation of a single stiffness, providing a microenvironment that simulates the anisotropy of the *in vivo* matrix.	Biologically and mechanistically validated	Patient-specific drug testing	137, 138, 141
Microfluidic systems	Enabling precise control over fluid dynamics, microscale spatial organization, and mass transport.	Microfluidic flow-rate and shear-stress characterization	Tumor-transendothelial migration-on-a-chip (TEMOC)	Simulates the transport and perfusion of fluids within the body, and is compatible with automation and high-throughput drug screening.	Biologically validated	Tumor cell interaction and drug-response modeling	123, 146
Mechanical actuation platforms	Actively apply external physical stimuli (such as compression, stretching, shear force, acoustic stimulation)	Compression testing, stiffness characterization, and acoustic actuation parameters	Acoustic Virtual 3D Scaffold (AV-Scaf); Compression-induced migration model.	Exploring how externally applied dynamic mechanical stress actively influences tumor behavior.	Biologically and mechanistically validated	Drug-response modeling	153, 155, 157
Multicellular reconstructive models	Co-culturing with relevant cells establishes a cell-driven TMME	Not routinely characterized	Co-culture of tumor organoids with fibroblasts (CAF), mesenchymal stromal cells (MSC), and immune cells.	Reconstruction of the mechanical microenvironment to generate and perceive the interactions among cell populations.	Biologically validated	Immunotherapy and drug-response modeling	161-164
3D bioprinting	Spatial programming of cells, bioinks and their associated mechanical properties to recapitulate heterogeneity.	Rheological and compressive mechanical testing	Extrusion-based, multi-nozzle 3D bioprinting; Machine learning-assisted frameworks.	The controllable and reproducible fabrication of tissue constructs possessing well-defined boundaries and mechanical gradients.	Biologically validated	Drug-response modeling	167, 168, 173, 174

## Abbreviations

TMME: tumor mechanical microenvironment
ECM: extracellular matrix
PDOs: patient-derived tumor organoids
TME: tumor microenvironment
3D: three-dimensional
IFP: interstitial fluid pressure
2D: two-dimensional
NAMs: new approach methodologies
CAFs: cancer-associated fibroblasts
MMP: matrix metalloproteinase
BM: basement membrane
FAK: focal adhesion kinase
EMT: epithelial-mesenchymal transition
DCs: dendritic cells
CSC: cancer stem cell
CRC: colorectal cancer
PEG: polyethylene glycol
PDS: patient-derived decellularized scaffolds
TEMOC: tumor-transendothelial migration-on-a-chip
PDMS: polydimethylsiloxane
CFD: computational fluid dynamics
FEM: finite element modeling
GelMA: methacrylate gelatin
PEGDA: polyethylene glycol diacrylate
AV-Scaf: acoustic virtual 3D scaffold
MSCs: mesenchymal stromal cells
PIC: polyisocyanopeptide
